# Learning to read as the formation of a dynamic system: evidence for dynamic stability in phonological recoding

**DOI:** 10.3389/fpsyg.2014.00660

**Published:** 2014-07-03

**Authors:** Claire M. Fletcher-Flinn

**Affiliations:** College of Education, University of Otago, DunedinNew Zealand

**Keywords:** dynamic systems, dynamic stability, theories of reading, reading acquisition, precocious reading, phonological recoding

## Abstract

Two aspects of dynamic systems approaches that are pertinent to developmental models of reading are the emergence of a system with self-organizing characteristics, and its evolution over time to a stable state that is not easily modified or perturbed. The effects of dynamic stability may be seen in the differences obtained in the processing of print by beginner readers taught by different approaches to reading (phonics and text-centered), and more long-term effects on adults, consistent with these differences. However, there is little direct evidence collected over time for the same participants. In this study, lexicalized (implicit) phonological processing, and explicit phonological and letter-sound skills are further examined in a precocious reader whose early development at 3 and 5 years has been extensively described (Cognition, 2000, 2004). At ages 10 and 14 years, comparisons were made with these earlier reports and skilled adult readers, using the same tasks for evidence of changes in reading processes. The results showed that along with an increase of reading accuracy and speed, her pattern of lexicalized phonological responses for reading did not change over time. Neither did her pattern of explicit phonological and letter-sound skills, aspects of which were inferior to her lexicalized phonological processing, and word reading. These results suggest dynamic stability of the word reading system. The early emergence of this system with minimal explicit skill development calls into question developmental reading theories that require such skills for learning to read. Currently, only the Knowledge Sources theory of reading acquisition can account for such findings. Consideration of these aspects of dynamic systems raise theoretical issues that could result in a paradigm shift with regard to best practice and intervention.

## INTRODUCTION

Children learn to read by forming links between mental representations of visual symbols (letters) in print words, and their pre-existing phonological (sound) and semantic (comprehension) representations for spoken language. The challenge for developmental theories of reading is to propose how such a reading system of connected representations might be formed. The purpose of this study was to consider learning to read as the formation of a dynamic system, and to test the concept of “dynamic stability” by examining behavioral data from a precocious reader for changes in her processing of print over time. Although such cases in the population are somewhat rare (1-3.5%, Jackson, 1992), it does not necessarily follow that the cognitive processes of learning to read in precocious readers, or their reading system components are dissimilar to other normal-progress readers. Most theories of reading make this “similarity” assumption and apply it to another small population, those having reading acquisition difficulties (3 to 10%, Snowling, 2013). According to Jackson and Coltheart (2001, p. 156), referring to precocious readers, even a single case provides unique “…opportunities to test hypotheses about conditions that are necessary for successful reading acquisition.” At the very least, such cases can contribute to the demarcation of limits on the range of application of current theories.

### CONNECTIONIST VIEWS OF READING

Current views of word reading, such as those from connectionist frameworks (e.g., Hutzler et al., 2004; Powell et al., 2006) assume neural system dynamics (analogous to computation in the brain), and address the general issue of how orthographic (print word) inputs are mapped onto spoken language (phonological) processes. These computer models (neural nets) have been applied to simulate the formation of a reading system in the brain through an initial architecture, and an extremely large corpus of words, input trials (exposures), learning rules, and error feedback. The initial architecture is changed as a result of these experiences, and the implemented reading model is compared with predictions from theories that are based on behavioral studies, and neuropsychological evidence on brain function. Although these connectionist models are purported to represent children’s capacities and knowledge of reading at specific points in their learning, they are at best only approximations (Seidenberg, 2007, p. 3), and perhaps, not surprisingly, they have been criticized for lacking in developmental plausibility (Cassidy, 1990; but see Seidenberg, 2007), and ecological validity (Hutzler et al., 2004).

Notwithstanding skepticism about their utility, several theoretical aspects of these connectionist models may be relevant for gaining a deeper understanding and contribute to an explanation of how children learn to read. The basic notion of an *emergent* (or self-organizing) process based on the interaction of simple components that gradually results in relatively robust complex structure is central to connectionist theory. The initial probabilistic outputs of connectionist reading models increasingly reflect the statistical structure of the word-training corpus as the system becomes fully trained and implemented. Such learning occurs by modifying the connectivity between processing units according to a weight adjustment algorithm as a function of the word-training experience. However, due to the provision of *supervised* training (of the target responses), as well as the initial pre-training on grapheme-phoneme correspondences of some models (Perry et al., 2007), most connectionist models are only “superficially” self-organizing, and are embedded with theoretical assumptions about how children learn to read. Two models (Dufau et al., 2010; Glotin et al., 2010) more closely resemble emergent principles and use developmentally appropriate lexical databases and unsupervised training. The principle of self-organization without top-down (deterministic) direction has been claimed as ubiquitous in biological development (van Geert, 2008) and in our natural (McClelland et al., 2010) and social environments (Bronfenbrenner, 1979).

### THEORIES OF LEARNING TO READ

In contrast to these connectionist theories, the focus of standard developmental theories of learning to read (e.g., Ehri, 1999, 2005) have been on the explicit skills claimed necessary for beginning reading, in particular, phonemic awareness and knowledge of letter-sound correspondences. These skills are used when a child attempts to read an unfamiliar word through a taught heuristic known as non-lexical (explicit) phonological recoding, in which each letter-sound is pronounced in sequence and with the deletion of unnecessary vowel sounds, they are recombined into a word (e.g., “ba – aa – ga,” for *bag*). The degree to which these explicit skills have been learnt forms the basis for summary (descriptive) performance accounts of the initial stages, or phases of the developmental theories. Share (1995) further emphasized the importance of non-lexical phonological recoding by claiming that it was a “self-teaching” device with successful attempts at recoding enabling the acquisition of word-specific orthographic knowledge required for skilled reading. Although concerned with learning, standard theories of reading have little to say about the dynamics of an emergent reading system, as children need to be first taught (explicitly) the basic skills, which are related but beyond the system (Jackson and Coltheart, 2001). These theories are also limited in explanatory power as they have been developed and tested with children who are taught to read with a phonics approach (Ramus, 2004; Fletcher-Flinn and Thompson, 2010).

An alternative reading acquisition theory, Knowledge Sources theory, also based on behavioral evidence, does share some principles of self-organization and implicit learning (Thompson et al., 1996; Thompson and Fletcher-Flinn, 2006, 2012). In this theory, it is claimed that sublexical patterns of print word input and corresponding information from the child’s phonological lexicon are induced implicitly as soon as the child attends to the relationship between letters and sounds within words, and a few words are stored in an orthographic lexicon (reading vocabulary with associated lexical meanings). These sublexical relations (ISRs) are induced from information across the child’s emerging reading vocabulary. They are used to generate responses to new words through lexicalized (implicit) phonological recoding, and the ISRs are continually updated as new words enter the child’s orthographic lexicon. Explicit skill learning is not necessary beyond a very rudimentary level. However, if such explicit skills are taught to children, they are considered another source of knowledge for the generation of responses to unfamiliar words.

Despite some shared concepts, Knowledge Sources theory differs from connectionist accounts in the specification of an orthographic lexicon. In connectionist accounts word knowledge is distributed and stored in the connections between the units rather than in an orthographic lexicon, resulting in the phenomenon of “catastrophic forgetting” (when new information interferes with old). Share, in his developmental theory, posits an orthographic lexicon from “self-learning,” contingent on a later major shift from reliance on non-lexical phonological recoding to lexical processes. This contrasts with Knowledge Sources theory in which learning to read is viewed as an emergent, dynamic and *continuous* process based on learning across phonological and orthographic lexicons. The orthographic lexicon for direct access to word representations (of both phonology and lexical meanings), and lexicalized phonological recoding processes based on ISRs are available soon after reading commences.

Evidence for the induction of ISRs has been accumulating and was shown experimentally for beginner readers of English (Thompson et al., 1996; Fletcher-Flinn and Thompson, 2000, 2004; Fletcher-Flinn et al., 2004), and more recently for the acquisition of a phonemic function of hiragana, a syllabic orthography, in beginner readers of Japanese (Fletcher-Flinn et al., 2014). These results indicate the possibility of a universal process of acquisition, which seems plausible if learning to read is the formation of a dynamic system (Seidenberg, 2011).

### THE DYNAMIC STABILITY OF A READING SYSTEM

Another general property of dynamic systems, important for theories of the acquisition of reading, is *dynamic stability*, in which patterns once acquired, are not easily modified or perturbed. According to Rolls (2012), recurrent (input) patterns promote stability, and *attractor* networks in the brain (neurons that collectively settle into stable patterns of firing) enable memories to be stored and recalled. These “integrate-and-fire” neural nets, when modeled in real continuous time, have the advantage of very fast recall. The gradual incremental changes (short-term dynamics) of the connection weights of an attractor network determine the final steady state of the connectionist system (long-term dynamics) (Munakata and McClelland, 2003; van Geert, 2008).

Dynamic stability is not an aspect that is within the scope of standard developmental theories of learning to read, insofar as their focus is on broad phases (e.g., Ehri, 1999, 2005) or changes in processing (Share, 1995). However, it has been considered by Knowledge Sources theory with regard to the developmental continuity of lexicalized phonological recoding for Maxine, a precocious reader who has been extensively studied from prior to the age of 2 years (Fletcher-Flinn and Thompson, 2000) and continued until the age of 7 years (Fletcher-Flinn and Thompson, 2004). It was shown that her processing of lexicalised phonological recoding was developmentally stable from 3 to 5 years of age, encompassing word-reading levels from 8 to 14 years (Fletcher-Flinn and Thompson, 2004).

With regard to normal-progress children learning to read, the long-term effects of dynamic stability may be seen from differences obtained when comparing the processing of print by 6-year-old beginner readers making normal-progress (Connelly et al., 2001) with phonics and text-centered approaches, as well as those 6- to 7-year-olds making slower progress (Thompson et al., 2008). For the same word reading ability, these studies showed faster text reading speed by beginners with non-phonics approaches compared with those children taught phonics. The latter were better able to read pseudowords, but were disadvantaged when reading low frequency words, and words that were irregular (Connelly et al., 2001). The speed of text reading advantage for non-phonics approaches was attributed to the greater time made available for text reading. Moreover, with the speed advantage, for an equal amount of time, beginners are exposed to more print words (and associated meanings).

Processing differences were also found among skilled adult readers of equivalent reading ability after nearly two decades beyond their initial reading instruction (Thompson et al., 2009). The adults who had initial instruction in phonics performed better on metalinguistic and letter-sound tasks, but similar to the children in the previous studies, they made more errors (regularizations) on contextually dependent pseudowords, and some low-frequency words than those without such instruction. It was suggested that the initial years of phonics reading instruction left a cognitive bias in processing associated with non-lexical phonological recoding that did not attenuate, or become superseded over time.

While the cross-sectional studies are intriguing because the results suggest a degree of long-term dynamic stability in the processing of print from different teaching approaches, they do not directly address questions of developmental change over time, or the stability of procedures for reading unfamiliar words. The purpose of this study was to examine these issues with regard to the learning and stability of lexicalized phonological processing in reading acquisition during development, alongside the learning of explicit phonological and letter-sound skills. This study examined Maxine’s reading development at age 10 and 14 years making comparisons with earlier published reports with the same tasks for evidence of changes in reading processes. Comparisons were made, where appropriate, with published results of skilled adult readers (Thompson et al., 2009) who, like Maxine, had not received explicit phonics instruction as beginner readers. This reading-level match was used to examine the extent to which the operation of components of her reading system differed from other highly skilled readers.

## SCHOOL EXPERIENCE AND INTERESTS, WORD READING ACHIEVEMENT, AND EXPLICIT PHONOLOGICAL SKILLS

Maxine entered a private intermediate school at the age of 8 years, and graduated from high school at 14 years. She studied the normal New Zealand curriculum, and continued with her musical, and other interests. She particularly enjoyed playing Pokemon on her Gameboy, skiing, and chess. Her school report at the end of intermediate school indicates that she was an exemplary student, with impressive examination grades well above the median in all major subjects. Other comments included her consideration of others, valued contributions to group discussions, and that she was well liked by students and staff. At her high school graduation, she won the class award for English Literature.

Maxine was assessed at two time periods, from 10 years 10 months (10:10) to 10:11, and 13:11 to 14:0 on a range of reading and phonological awareness tests. Informed written consent was obtained from Maxine and her parents, and the study was approved by the Auckland Human Subjects Ethics Committee, as part of a larger study on precocious readers. Standardized word reading tests included the Wide Range Achievement Test 3 – Combined Form (WRAT-3, Wilkinson, 1993) for both oral reading and spelling, and the Nelson-Denny Reading Test [N-D, Brown et al. (1981)], Vocabulary subtest, to assess reading comprehension of single words. These assessments showed that Maxine continued her precocious word reading development, reaching beyond high school levels by 10:11 on the WRAT-3 oral reading subtest, and by 13:11 she was equivalent to the comparison sample of adults on the N-D Vocabulary subtest (Thompson et al., 2009). Spelling was consistent with her reading ability.

The phonological awareness tasks included the Rosner Test of Auditory Analysis Skills (Rosner and Simon, 1971), and the Yopp–Singer phoneme segmentation task (Yopp, 1988). The Rosner Test, consisting of 40 items, assesses the skill of the deletion of phonemes from various positions in words, e.g., saying *man* without the “m” sound. The Yopp-Singer phoneme segmentation task requires the pronunciation of sounds of spoken words in the correct order, e.g., “Tell me all the sounds that you can hear in the word ‘dog”’ (Three sounds relating to the three phonemes comprise the correct response.) Both tests had been administered when she was 5 and 7 years (but in a 13 item version of the Rosner Test at 5 years, Fletcher-Flinn and Thompson, 2004). The Rosner Test placed Maxine at the Grade 6 (U.S.) ceiling level of the test from 7 years (**Table 1**). On the Yopp-Singer segmentation task, for the same ages, she was within (or close to) +1 standard deviation (SD) of the norms based on children in kindergarten (average age of 5:10) in U.S. schools with some letter-sound instruction. Their average score was 54% (SD 35%). Although she attained 92% correct at 13:11, her performance on this task continued to be underdeveloped relative to her age and reading ability.

**Table 1:** Test age levels for phonological awareness for Maxine at chronological ages 7:3 to 13:11, and mean percentage correct on phoneme awareness and the extended Scarborough task for Maxine and New Zealand university students without phonics instruction (standard deviation in parenthesis).

	Chronological age (years and months)			
	Maxine			NZ university students
	7:3–7:11	10:11	13:11	
**Test ages**
Yopp-singer phoneme segmentation	5	5	5	–
Rosner deletion	11	11	11	–
**Percentage correct**
Phoneme awareness (aural)	–^a^	53	70	61 (11)
**Extended Scarborough task**
Graphophonemic awareness	–	63	77	61 (20)
Graphophonemic segments	–	43	53	39 (13)

a Tests not administered.

As Maxine was reading at the same level on the N-D reading test as the comparison sample of adults, the same phonemic awareness and graphophonemic (extended Scarborough task, Thompson et al., 2009) measures used for them were administered to her. In the phoneme awareness task 30 words were presented in aural form and the task was to count the number of the “smallest sounds” in each word, e.g., four for *socks*. The same words from this task were used in a graphophonemic task in which the words were presented in print form. In this task, the participant must read the word, note the number of sounds in the word (awareness score), and underline the letter or letter sequence belonging to each sound (identity score). At 10:11, Maxine’s accuracy on tests of phoneme awareness, graphophonemic awareness, and graphophonemic segments were all within 1 SD of that reported for the adults. She showed continued development of these skills at 13:11, although remaining within about 1 SD of the adult means for these tasks (**Table 1**).

It seems fair to say that as Maxine’s word reading advanced, her phoneme awareness skills continued to develop and were not markedly different to skilled adults at the same reading level. However, her explicit skill at segmenting spoken words into phonemes and to recite them in order remained underdeveloped and consistent with her earlier kindergarten level performance on this task. This is interesting because Yopp (1988) found phoneme deletion tasks, like the Rosner Test on which Maxine performed adequately, to be more difficult than phoneme segmentation tasks, such as the Yopp-Singer, for his sample of 5-year-olds. Although modeled occasionally by her parents, the “sounding-out” response heuristic for this task was never used by Maxine (Fletcher-Flinn and Thompson, 2000, p. 184), and is not a skill that would have been taught to the New Zealand adults (Thompson et al., 2009). It is, however, an explicit skill that is required for non-lexical phonological recoding to read unfamiliar words.

## EXPERIMENT 1: EXPLICIT LETTER-SOUND SKILLS

The set of three tasks – letter naming, letter sounds, and digraph sounds – which were employed for Maxine (Fletcher-Flinn and Thompson, 2004), and for the comparison group of adults (Thompson et al., 2009) were administered when Maxine was 10:10 and 13:11 using the same procedure with computer presentation in lowercase. Speeded response instructions were given and there was no correction of responses. In scoring, correct letter-sound responses were those taught in explicit phonics instruction. For the 29 digraphs, (e.g., *ee*, *aw*, *ch*), Maxine was instructed to pronounce the sound associated with the two letters, and scoring was the same as in the earlier studies.

Maxine’s mean accuracy (**Table 2**) for giving phonic sounds to letters and digraphs from 5:8 to 13:11 was within (or close to) 1 SD of the adult comparison sample (Thompson et al., 2009). There was no significant increase in her mean percentage accuracy over time using the McNemar test for the significance of changes, from 5:8 to 10:10 for giving phonic sounds to letters, *X*^2^(1) = 1.54, and digraphs, *X*^2^(1) = 1.23, *p* > 0.20; or, from 10:10 to 13:11 for giving phonic sounds to letters, *X*^2^(1) = 0.0, and digraphs *X*^2^(1) = 0.16, *p* > 0.68. Similarly, over the longer span of time from 5:8 to 13:11 there was no significant change in percentage accuracy for giving phonic sounds to letters, *X*^2^(1) = 1.54, *p* = 0.21, or digraphs *X*^2^(1) = 2.5, *p* = 0.11.

**Table 2:** Experiment 1: Mean percentage accuracy and response times (RTs) for lowercase letter names, letter sounds, and digraph sounds for Maxine at 5:8, 10:10, and 13:11, and for the NZ university students without phonics instruction (standard deviation in parenthesis).

	Letter names	Letter sounds	Digraph sounds
**Mean percent correct**
Maxine at 5:8	92	65	62
Maxine at 10:10	100	81	76
Maxine at 13:11	100	81	82
NZ university students	100 (0)	75 (12)	73 (10)
**Mean RTs (ms) for correct responses**
Maxine at 5:8	522	551	558
Maxine at 10:10	551	765	683
Maxine at 13:11	638	651	589

A repeated-measures ANOVA over items for Maxine’s response times (RTs) on letter names to which she responded accurately showed a significant change over time *F*(2,38) = 10.45, *p* < 0.0001, η^2^ = 0.36. Using paired *t*-tests, her performance at 13:11 was slower than at 5:8, *t*(19) = -5.69, *p* < 0.0001; and 10:10, *t*(24) = -4.04, *p* < 0.0001. There was no change from 5:8 to 10:10, *t*(20) = -1.19, *p* = 0.25. The results were similar for responses to letter sounds, *F*(2,22) = 12.01, *p* < 0.0001, η^2^ = 0.52, with significantly slower RTs from 5:8 to 13:11, *t*(12) = -4.65, *p* < 0.001; and 10:10, *t*(12) = -3.87, *p* < 0.002. She was somewhat faster from 10:10 to 13:11, *t*(12) = 2.50, *p* < 0.02. Responses to digraphs also changed significantly over time, *F*(2,16) = 9.27, *p* < 0.002, η^2^ = 0.54, with slower RTs from 5:8 to 10:10, *t*(9) = -4.08, *p* < 0.003, but faster from 10:10 to 13:11, *t*(20) = 2.64, *p* < 0.02. There was no difference in RTs for digraphs at 5:8 and 13:11, *t*(8) = -1.99, *p* < 0.08, although it was in the negative direction, indicating a decrease in speed with age.

In summary, Maxine’s mean percentage accuracy for providing phonic sounds to letters and digraphs was similar to the comparison sample of adults, and did not change significantly over time. Similar to Maxine, the adults had not experienced explicit phonics instruction as beginner readers, and their mean accuracy did not exceed 75% (SD = 12). Of interest, Maxine showed a tendency for slower RTs with age for letter names, sounds, and digraphs compared with her performance at 5:8.

## EXPERIMENT 2: NONWORD PRONUNCIATION

Two nonword pronunciation tasks were administered to Maxine. Each task consisted of sets of Regular, body-consistent; Regular, body-inconsistent; and Irregular, body-consistent nonwords presented in a randomized sequence. The first source of these nonwords was from Andrews and Scarratt (1998, Experiment 2), and the second was from Coltheart and Leahy (1992, Task 2). The first category required a regular response for accuracy (e.g., *stell*, *dilt*). The other two categories were heterophonic nonwords. For the category of nonwords with Inconsistent lexical bodies (e.g., *dush* which can be pronounced with –*ush* as in “rush,” or “push”) either the regular, or irregular pronunciation, respectively, was acceptable. The first two categories of nonwords consisted of 40 and 20 items, respectively, from each source. The third classification consisted of nonwords that always have Irregular lexical bodies (e.g., *thild*) that occur in several real words (e.g., *child*). There were 20 of these nonwords in the Coltheart and Leahy task, and 24 of them in the other source. Andrews and Scarratt (1998) also included 24 Irregular Unique nonwords (e.g., *hourt*, *yign*) with Irregular lexical bodies having only one real word exemplar. The procedure was the same as in the previous reported experiments (Fletcher-Flinn and Thompson, 2000, 2004; Thompson et al., 2009^1^).

### ANDREWS AND SCARRATT NONWORDS (1998)

**Table 3** shows the percentages of regular and irregular responses for the categories of nonwords on the Andrews and Scarratt (1998) task. Overall mean combined accuracy scores were calculated, which included: (1) the *regular* responses to the Regular Consistent and the Inconsistent nonwords, and (2) the *irregular* responses to the Irregular consistent and Irregular Unique nonwords. (The regular pronunciations to these two categories were excluded as less accurate “regularizations.”)

**Table 3:** Experiment 2: Mean percentage of regular and irregular pronunciations for Andrews and Scarratt nonwords varying in regularity and consistency of body spelling for Maxine at 5:9, 10:10, and 14:0, and for the New Zealand university students without phonics instruction (standard deviation in parenthesis).

	Regular consistent	Inconsistent	Irregular consistent	Irregular unique
**Regular pronunciations**
5:9 years	95	88	4	25
10:10 years	98	83	0	17
14:0 years	93	90	4	21
New Zealand university students	92 (4)	84 (7)	10 (9)	37 (10)
**Irregular pronunciations**
5:9 years	–^a^	10	83	63
10:10 years	–	15	92	71
14:0 years	–	10	88	75
New Zealand university students	–	10 (6)	63 (17)	46 (8)

a Dashes indicate that regular pronunciations do not exist for regular consistent nonwords.

The combined mean percentage accuracy for *regular* responses to the Regular Consistent and the Inconsistent nonwords (**Table 4**) showed that Maxine varied very little (between 92 and 91%) from 5:9 to 14:0. This was within +1 SD of the mean percentage accuracy^1^ for the adults without phonics instruction. Accurate combined mean percentage accuracy for *irregular* responses to the Irregular consistent and Irregular Unique nonwords was 82% for Maxine at both 10:10 and 14:0, and at 5:9, it was 73%. She exceeded the mean percentage accuracy of the adults by +2.2 SD. The McNemar test showed no significant change in irregular responses for Maxine from 5:9 to 10:10 and 14:0, *X*^2^(1) = 1.22, *p* = 0.27.

**Table 4:** Experiment 2: Mean percentage of regular and irregular pronunciations for Coltheart and Leahy nonwords varying in regularity and consistency of body spelling for Maxine from 3:4 to 14:0, and New Zealand university students without phonics instruction (standard deviation in parenthesis).

	Regular consistent	Inconsistent	Irregular consistent
**Regular pronunciations**
Maxine at 3:4	100	55	10
Maxine at 5:5	100	65	30
Maxine at 11:10	100	90	10
Maxine at 14:0	100	90	5
New Zealand University students	95 (6)	71 (13)	28 (12)
**Irregular pronunciations**
Maxine at 3:4	–^a^	35	80
Maxine at 5:5	–	30	70
Maxine at 11:10	–	10	70
Maxine at 14:0	–	10	75
New Zealand university students	–	21 (13)	46 (16)

a Dashes indicate that irregular regular pronunciations do not exist for regular consistent nonwords.

Response times were analyzed for those response categories above mean acceptable response rates of 33% or higher. At 5:8, 10:10, and 14:0, Maxine’s mean RTs for accurate *regular* responses for the Regular Consistent nonwords were: 549, 497, and 380 ms; and, for the Inconsistent nonwords 555, 491, and 376 ms, respectively.

For accurate regular responses to Regular consistent nonwords, a repeated-measures ANOVA by items showed a significant change in RTs over time, *F*(2,68) = 53.97, *p* < 0.0001, η^2^ = 0.61. Paired *t-*tests indicated that Maxine was significantly faster with each age comparison: 5:9–10:10, *t*(37) = 2.29, *p* = 0.03; 10:10–14:0, *t*(35) = 8.17, *p* < 0.0001; and, 5:9 to 14:0, *t*(35) = 11.21, *p* = 0.0001. A similar pattern was shown by Maxine on correct regular responses to Inconsistent regular nonwords, *F*(2,52) = 46.57, *p* < 0.0001, η^2^ = 0.64 with faster RTs with age: 5:9–10:10, *t*(27) = 2.02, *p* = 0.05; 10:10–14:0, *t*(31) = 9.30, *p* < 0.0001; and, 5:9–14:0, *t*(29) = 11.45, *p* = 0.0001.

For correct *irregular* responses, Maxine’s mean RTs for the same ages, for Irregular Consistent nonwords were: 553, 534, and 383 ms; and, for Irregular Unique nonwords: 582, 562, and 374 ms. There was a significant change in RTs over time for irregular responses to Irregular Consistent nonwords, *F*(2,34) = 32.24, *p* < 0.0001, η^2^ = 0.66. She was significantly faster at 14:0 than at 10:10, *t*(19) = 9.19, *p* < 0.0001; and, at 14:0 than at 5:9, *t*(18) = 7.43, *p* = 0.0001. RTs were equivalent at 5:9 and 10:10, *t*(17) = 1.29, *p* = 0.22. The irregular responses to Irregular Unique nonwords showed a similar pattern of change, *F*(2,20) = 36.75, *p* < 0.0001, η^2^ = 0.79. There was no difference in RTs at 5:9 and 10:10, *t*(10) = 1.208, *p* = 0.30, but she was significantly faster at 14:0 than at 10:10, *t*(14) = 6.85, *p* < 0.0001; and, at 14:0 than at 5:9, *t*(12) = 7.14, *p* = 0.0001.

### COLTHEART AND LEAHY NONWORDS (1992)

Maxine’s mean percentage accuracy for *regular* responses to regular consistent and inconsistent nonwords, averaged over the two categories of nonwords, at both 10:10 and 14:0 was 95% (**Table 4**), which was within 1 SD of the comparison sample of adults at 83%. Accurate irregular responses to the Irregular Consistent nonwords were 80% at 3:4, and 70% at 5:5, with these changes reported not significant (Fletcher-Flinn and Thompson, 2004). Maxine’s accurate irregular responses for the same set of nonwords were 70% at 10:10, and 75% at 14:0. She was, respectively, +1.5 SD and +1.81 SD more accurate than the comparison sample of adults. The McNemar test for the significance of change for each age comparison was not significant, *X*^2^(1) < 1.

Maxine’s response times were analyzed for those categories above mean acceptable response rates of 33% or higher. At 5:8, 10:10, and 14:0, respectively, Maxine’s mean RTs for regular responses to regular consistent nonwords were: 560, 477, 386 ms, and for ambiguous inconsistent nonwords: 609, 525, 412 ms. Her mean RTs for irregular responses to Irregular Consistent nonwords were: 584, 501, and 409 ms. An ANOVA with two factors (time, category type) over items was significant, for time, *F*(2,139) = 78.76, *p* < 0.0001, η^2^ = 0.53, and category type, *F*(2,139) = 4.48, *p* < 0.013, η^2^ = 0.06. There was no interaction, *F* < 1. Paired *t*-tests with Bonferroni adjustments showed that Maxine was faster at 14:0 compared with her earlier responses at 5:9 and 10:10, MSE = 4951.90, *p* < 0.0001; and she gave regular responses to Regular consistent nonwords faster than to Ambiguous inconsistent nonwords, MSE = 4770.36, *p* < 0.03. There was no difference in RTs for irregular responses to Irregular consistent nonwords compared with the regular responses for the other two categories, MSE = 4770.36, *p* ≤ 1.

### DISCUSSION

Although Maxine’s ceiling level of performance on the Andrews and Scarratt nonwords from 5:9 did not leave much room for gains in accuracy, she was equivalent to the adult comparison sample for regular responses to the regular consistent and inconsistent nonwords, and she exceeded them on the irregular responses to the irregular consistent and irregular unique nonwords. The speed of Maxine’s responses increased significantly over time, and by 14 years her mean RT was 378 ms, over the four categories of items, which exceeds the mean RT of 639 ms of the university students from the Andrews and Scarratt’s (1998, Table 8) study. Maxine’s accuracy on the Coltheart and Leahy (1992) nonwords showed the same pattern of performance for regular and irregular responses, and the same decrease in RTs with age. Similarly, by 14 years, with a mean RT (over categories) of 402 ms, she exceeded the mean RT of 704 ms for the university students from Coltheart and Leahy (1992, Table 4).

The results for Maxine on the two nonword tasks converge to indicate a high degree of proficiency of phonological recoding, relative to samples of skilled adult readers. However, for the present purposes, the most important aspect of her performance was that she retained the same pattern of category responses over time. Lexical phonological processing was developmentally stable from 3 to 14 years of age, covering word-reading levels from 8 years to skilled adult levels of performance. Concomitantly, her speed of processing nonwords continued to increase over this period of time.

## EXPERIMENT 3: WORD NAMING AND LEXICAL SEMANTIC INFLUENCES

Strain et al. (1995, Experiment 2) showed that for adults the degree of imageability, which is a semantic characteristic of words, contributes to word reading accuracy when the words are of low frequency and irregular in spelling. The same result was found for Maxine at 5:9 years and an 11-year-old matched word-reading level comparison sample of normal progress readers without phonics instruction (Fletcher-Flinn and Thompson, 2004), and for the comparison sample of adults (Thompson et al., 2009).

At 10:10 and 14:0, the words from Strain et al. (1995) were administered to Maxine with the same presentation and scoring procedure as for the previous experiments with the 11-year-olds and adults. The stimuli comprised four categories of low-frequency words, with 16 words in each: irregularly spelt words of high imageability (e.g., *boulder*, *climb*), irregularly spelt words with low imageability (e.g., *broader*, *cache*), regularly spelt words of high imageabilty (e.g., *banner*, *cliff*), and regularly spelt words with low imageability (e.g., *blessing*, *cleft*).

Maxine showed the same ceiling level of accuracy as the adults for regular words and for words of high imageability by 10:10 (**Table 5**), and for irregular words of low imageability by the age of 14 years. Similar to Maxine’s performance at 5 years, using McNemar’s test, regular words were read more accurately than irregular words at 10:11, *X*^2^(1) = 4.94, *p* < 0.03, but not at 14:0, *X*^2^(1) < 1. There was no effect of imageability at either age 10:11 or 14:0, *X*^2^(1) = 2.52, *p* = 0.11, and *X*^2^(1) < 1, respectively. Similar to her previous performance at 5:9 and the adults, regularization responses accounted for 83 and 100% of her errors on the low imageability words with irregular spellings at 10:10 and 14:0, respectively.

**Table 5:** Experiment 3: Mean percent correct and RTs for words varying in regularity of spelling and in imageability for Maxine at 5:9, 10:10 and 14:0, and New Zealand university students without phonics instruction (standard deviation in parenthesis).

	Regular		Exception	
	High imageability	Low imageability	High imageability	Low imageability
**Mean percentage correct**
Maxine at 5:9	94	94	88	50
Maxine at 10:10	100	100	94	63
Maxine at 14:0	100	100	100	88
NZ university students	100 (0.0)^a^	99 (2.9)	98 (4.2)	83 (10.2)
**Mean response time**
Maxine at 5:9	481	506	492	493
Maxine at 10:10	493	520	454	474
Maxine at 14:0	418	409	407	444
NZ university students (without phonics instruction)	561 (79)	593 (87)	581 (81)	642 (110)

At 14:0, Maxine’s mean RTs were 1.8–2.15 SD shorter than the adults across all four categories of words. In a 3-way ANOVA by items, there was a significant effect of age, *F*(2,157) = 17.20, *p* < 0.0001, η^2^ = 0.18, but no effect for regularity or imageability, or any interaction (*p* > 0.10). Paired *t*-tests with Bonferroni adjustments on the main effect of time showed that Maxine was faster at 14:0 compared with her earlier responses at 5:9 and 10:10 (MSE = 4770.36, *p* < 0.0001).

These results are consistent with the standardized word reading assessments. She reached adult (university) levels of accuracy by 14 years, with significantly faster RTs to isolated words than the adults. Although there was similarity on this task to some aspects of her earlier performance, the absence of any differences in accuracy or RTs for the effects of regularity or imageability may be attributed to reaching ceiling levels of performance. In that case, the phonological processing of words is too efficient to be assisted by a word’s semantic characteristics.

## GENERAL DISCUSSION

Although Maxine continued to develop greater speed and word reading accuracy over time, her pattern of responses for categories of nonwords did not change from when she was much younger. According to Fletcher-Flinn and Thompson (2000), Maxine was able to read nonwords while having underdeveloped explicit skills for word reading when she was 3 years by inducing sublexical relations (ISRs) between orthographic and phonological components in words that are stored in orthographic memory. Through a process of lexicalized phonological recoding, she was able to use these ISRs, in turn, to read unfamiliar words. The current findings suggest that the processes associated with lexicalized phonological recoding apparently become stable very early in acquisition, and are resistant to change. This long-term stability is not within the explanatory range of the standard developmental theories of learning to read that propose phases (e.g., Ehri, 1999, 2005), or shifts in phonological recoding processes (Share, 1995). It is, therefore, a limitation of these theories.

As Maxine continued to develop her precocious reading skills, both word reading and word comprehension, going well beyond the normal attainment for her age, her phonological awareness showed differential success. Her performance was age appropriate on a test of phoneme deletion, and at 10:11 and 13:11 equivalent to the comparison sample of adults without phonics instruction on an aural phoneme awareness task and two graphophonemic tasks. However, on another test (Yopp-Singer) involving segmentation and pronunciation of phoneme components of words, she only reached the 5-year-level of normal age controls, which is consistent with her underdeveloped performance on this task when much younger (Fletcher-Flinn and Thompson, 2000, 2004). It seems reasonable to conclude that she had not learnt the explicit procedure for non-lexical phonological recoding, and hence, it remained underdeveloped.

Maxine’s proficiency in providing the phonic sounds for isolated letters, and sounds for digraphs was comparable to a sample of adults without phonics instruction whose learning was also incomplete, averaging 74% across the two categories (Thompson et al., 2009). In contrast to an increase of speed for word (and nonword) reading, her response to individual letters tended to become slower over time. Her mean response times for letter names, phonic sounds for letters and digraph sounds at 13:11 were 3.67 SD, 2.13 SD, and 1.99 SD longer, respectively, than her mean accuracy (combined over items) for the low frequency words of Strain et al. (1995). For comparison, the mean RTs for Maxine’s responses to these words, and the nonwords (combined over items) from Andrews and Scarratt (1998) were within 0.52 SD. The significant differences between Maxine’s response times for isolated letters, and word and nonword reading indicate that the reading system formed consists of word representations, and does not include explicit responses to isolated letters, which are considered extrasystem entities (Jackson and Coltheart, 2001, p. 103). Of more importance is the lack of any difference in response times between real words and nonwords, indicating that the source of knowledge for reading the nonwords must be lexical in the form of ISRs.

In summary, although showing more processing efficiency, Maxine’s pattern of performance was not different to the comparison group of adult readers, nor had it been different to earlier reading-age matches (Fletcher-Flinn and Thompson, 2000, 2004). The evidence presented on the long-term stability of lexicalized phonological recoding as shown by Maxine’s stable performance over time is indicative of the formation of an attractor state of a dynamic system displaying very fast recall. The reading system formed need not be underpinned by explicit skill knowledge as claimed by standard developmental theories (e.g., Share, 1995; Ehri, 1999, 2005), as lexicalized procedures appear to be sufficient for both the establishment, with minimal skills (Fletcher-Flinn and Thompson, 2000), and the expansion and stability of the emergent system, as shown by these results. These findings support Knowledge Sources theory and converge with earlier cross-sectional studies on the induction of ISRs in normal-progress readers without phonics instruction (Thompson et al., 1996; Fletcher-Flinn and Thompson, 2000, 2004), those with such instruction (Fletcher-Flinn et al., 2004), and the long-term effects of differing approaches to reading in normal-progress readers (Connelly et al., 2001; Thompson et al., 2008), and skilled adults (Thompson et al., 2009).

The accumulating (see Thompson, 2014, for a review) and converging evidence from these studies indicates that non-lexical phonological recoding is not central to acquiring word-specific orthographic knowledge as Share (1995) claims, although if taught, successful recoding may contribute to the addition of new words in the orthographic lexicon (Thompson et al., 1996; Thompson and Fletcher-Flinn, 2006, 2012). It is interesting to speculate that in this case, if the procedural (instructional) heuristic is abstracted along with new word pronunciations, it might explain the irregular word disadvantage of beginner readers with phonics instruction (Connelly et al., 2001). Alternatively, with the emergent reading system achieving early stability, regularization of new words might be due to an excessive exposure to regular words (and pseudowords) from typical phonics programes, thus creating an initial bias in the formation of ISRs. A combination of both would strengthen any tendency toward the regularization of new words, leaving a long-term cognitive bias in processing (Thompson et al., 2009). Consideration of these questions and issues arising from them could provide the impetus for future research, and may pave the way for changes to best optimize reading instruction and intervention.

If scientific progress is to be made, then our current theories of how children learn to read need better scrutiny, with both intensive longitudinal study of single cases and population samples, to test and delineate their range of application. Ideas from connectionist theory, and more generally from cognitive science, on the formation of dynamic systems can contribute to these new tests.

## Conflict of Interest Statement

The author declares that the research was conducted in the absence of any commercial or financial relationships that could be construed as a potential conflict of interest.

## Footnotes

1 Only items that were acceptable in both standard New Zealand and Scottish accents were scored in Thompson et al. (2009, p. 227), whereas all items were scored for comparison with Maxine in this study.
